# 

**Published:** 1902-11-15

**Authors:** 


					﻿THE
DENTAL REGISTER
EDITED BY
NELVILLE S. HOFF, D. D.S.,
ANN ARBOR, MICH.
Vol. LVI. NOVEMBER 15,1 1902. i > ~~NoTIT.'
PRICE, ONE DOLLAR A YEAR IN ADVANCE.
PUBLISHED MONTHLY BY
SAMUEL· A, CROCKER & CO.,
THE OHIO DENTAL AND SURGICAL DEPOT.
to 30 W. “fit JSt., Cineiniiiiti, O.
Entered at the Postoffice at Cincinnati, 0., as second-class mail matter.
				

